# Optimization of Micellar Electrokinetic Chromatography Method for the Simultaneous Determination of Seven Hydrophilic and Four Lipophilic Bioactive Components in Three *Salvia* Species

**DOI:** 10.3390/molecules200815304

**Published:** 2015-08-21

**Authors:** Jiliang Cao, Ji Hu, Jinchao Wei, Baocai Li, Mi Zhang, Cheng Xiang, Peng Li

**Affiliations:** 1 State Key Laboratory of Quality Research in Chinese Medicine, Institute of Chinese Medical Sciences, University of Macau, Macau 999078, China; E-Mails: caojiliang126@126.com (J.C.); huji_cassie@163.com (J.H.); wjc551@sina.com (J.W.); 2 Faculty of Life Science and Technology, Kunming University of Science and Technology, Kunming 650500, China; E-Mails: kmzhangmi@163.com (M.Z.); chengxiang0871@126.com (C.X.)

**Keywords:** micellar electrokinetic chromatography, buffer modifiers, phenolic acids, tanshinones, *Salvia* species

## Abstract

A micellar electrokinetic chromatography (MEKC) method was developed for the simultaneous determination of seven hydrophilic phenolic acids and four lipophilic tanshinones in three *Salvia* species. In normal MEKC mode using SDS as surfactant, the investigated 11 compounds could not be well separated. Therefore, several buffer modifiers including β-cyclodextrins (β-CD), ionic liquid 1-butyl-3-methylimidazolium tetrafluoroborate ([bmim]BF4) and organic solvents have been added to the buffer solution to improve the separation selectivity. Under the optimized conditions (BGE, 15 mM sodium tetraborate with 10 mM SDS, 5 mM β-CD, 10 mM [bmim]BF4 and 15% ACN (*v*/*v*) as additives; buffer pH, 9.8; voltage, 20 kV; temperature, 25 °C), the 11 investigated analytes could achieve baseline separation in 34 min. The proposed MEKC was additionally validated by evaluating the linearity (R^2^ ≥ 0.9965), LODs (0.27–1.39 μg·mL^−1^), and recovery (94.26%–105.17%), demonstrating this method was reproducible, accurate and reliable. Moreover, the contents of the 11 compounds in three *Salvia* species, including *S*. *miltiorrhiza*, *S. przewalskii* and *S. castanea* were analyzed. The result showed that the established MEKC method was simple and practical for the simultaneous determination of the hydrophilic and lipophilic bioactive components in *Salvia* species, which could be used to effectively evaluate the quality of these valued medicinal plants.

## 1. Introduction

*Salvia*, one of the largest genera of the Lamiaceae family, is widely distributed in various regions of the world and is represented by over 1000 species [1]. Some members of *Salvia* plants are well noted for their therapeutic properties because of their diverse biological activities. For example, the dried root and rhizome of *S. miltiorrhiza* (known as danshen in Chinese), is one of the most frequently used traditional Chinese herbs in China, with beneficial effects on cardio-cerebrovascular diseases, hepatitis, hepatocirrhosis, chronic renal failure, dysmenorrhea, anti-tumor effects, as well as the neuroprotective and antiparkinsonian activity [2,3,4,5]. However, other species, such as *S. yunnanensis*, *S. przewalskii* and *S. castanea*, have been used as substitutes for danshen in some areas of China, especially in the southwest. Since the contents of chemical constituents usually differ in various *Salvia* species, the development of proper methods for determining the active components in danshen and related species is of significant importance for the rational utilization of these medicinal plants.

According to the results of pharmacological studies and chemical investigations on these *Salvia* species, there are mainly two types of bioactive components responsible for their remarkable pharmacological or therapeutic effects, phenolic acids (e.g., salvianolic acid B and rosmarinic acid) and tanshinones (e.g., tanshinone IIA and dihydrotanshinone I) [6,7,8,9,10,11,12]. Therefore, both hydrophilic phenolic acids and lipophilic tanshinones are often considered as chemical markers for quality evaluation of danshen and related medicinal plants [13,14,15]. For example, in Chinese Pharmacopoeia (edtion 2010) [16], hydrophilic salvianolic acid B and lipophilic tanshinone IIA are chosen together as chemical indicators to assess the quality of danshen.

Until now, a number of analytical methods, such as HPLC [13,17,18,19], ultra-performance liquid chromatography (UPLC) [20,21], or liquid chromatography-mass spectrometry (LC-MS) [15,22], have been established for simultaneous analysis of the hydrophilic and lipophilic components in danshen and its related preparations. These LC-based methods could easily achieve, not only satisfactory repeatability, but also high sensitivity. Meanwhile, they have also suffered from some disadvantages, especially the great consumption of organic solvents, even when using UPLC techniques. As an alternative, due to the advantages of short analysis time, high resolution, separation efficiency, and minimal consumption of sample and solvents [23], capillary electrophoresis (CE) has been introduced for the analysis of phenolic acids using the capillary zone electrophoresis (CZE) mode [24,25,26], and tanshinones using nonaqueous capillary electrophoresis (NACE) [27], and nonaqueous MEKC [28] or capillary electrochromatography (CEC) [29] modes in danshen. However, because of the great differences in the polarity of phenolic acids and tanshinones, only a few CE modes, such as microemulsion electrophoresis chromatography (MEEKC), have been successfully developed for the simultaneous determination of these two types of components [30].

**Figure 1:**
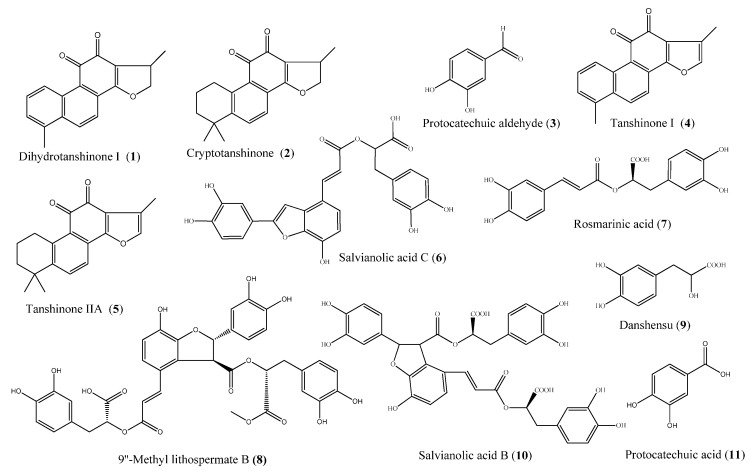
The chemical structures of 11 investigated compounds.

MEKC, an important branch of CE, has been widely employed recently due to its special ability in the separation of both neutral and charged compounds with high selectivity, micellar system stability, and easy handling [31]. In the present work, an MEKC method was developed to simultaneously separate seven hydrophilic phenolic acids, protocatechuic aldehyde (**3**), salvianolic acid C (**6**), rosmarinic acid (**7**), 9″-Methyl lithospermate B (**8**), danshensu (**9**), salvianolic acid B (**10**), and protocatechuic acid (**11**), and four lipophilic tanshinones, dihydrotanshinone I (**1**), cryptotanshinone (**2**), tanshinone I (**4**), and tanshinone IIA (**5**) (structures shown in Figure 1). Several buffer modifiers such as β-cyclodextrins (β-CD), ionic liquid 1-butyl-3-methylimidazolium tetrafluoroborate ([bmim]BF_4_) and organic solvents have been used to improve the separation selectivity. An optimization study was performed by assessing the effects of sodium dodecyl sulfate (SDS), organic solvents, [bmim]BF_4_, β-CD, and buffer pH. Furthermore, the contents of the 11 investigated analytes in three *Salvia* species, *S. miltiorrhiza*, *S. przewalskii* and *S. castanea*, were analyzed using the developed and validated MEKC method.

## 2. Results and Discussion

### 2.1. Preliminary Investigations

According to our previous work, the seven hydrophilic compounds could be perfectly separated under the ordinary CZE mode [24]. However, once the lipophilic compounds were included as target analytes, the baseline separation could not be achieved using CZE mode, even if different buffer compositions, concentrations, and additives were considered. A possible reason is that the four lipophilic analytes could not be charged in CZE buffers, resulting in their co-migration with the electroosmotic flow (EOF). Thus, as another operation mode of CE, MEKC has been considered since it can always complete the separation of both charged and neutral analytes. Initially, under normal MEKC conditions, without addition of any modifiers (SDS concentration 5–50 mM, sodium tetraborate concentration 5–40 mM and pH 8.5–11.0), no successful separation was obtained, especially, the four lipophilic analytes exhibited only a single peak. The poor selectivity and low resolution were possibly attributed to the high hydrophobicity of the four lipophilic components, which tended to be absorbed completely into the inner core of SDS micelles.

Therefore, a number of modifiers were further utilized to improve the selectivity of the MEKC system, including different organic solvents, the ionic liquid [bmim]BF_4_ and β-CD. In general, the addition of an organic solvent can increase the solubility of lipophilic analytes, reduce the EOF, and improve the separation efficiency. By adding ACN to the run solution, most of the 11 analytes could be well separated but, unfortunately, cryptotanshinone and tanshinone I still overlapped as one single peak. In MEKC, the separation mechanism of analytes is based on the differential partitioning between the micellar stationary phase and the aqueous phase. In this experiment, cryptotanshinone and tanshinone I might have had more similar partition coefficients in the micellar phase, which led to the co-elution of the two analytes, even when using ACN as the additive. Thus, [bmim]BF_4_ was further selected as a modifier to enhance the selectivity and the results showed a total baseline separation of the four lipophilic analytes. In addition, β-CD was also added to solve the co-elution problem of salvianolic acid C and rosmarinic acid.

### 2.2. Optimization of MEKC Conditions

According to the preliminary study, 15 mM sodium tetraborate was selected as the optimal BGE by a comprehensive consideration of the migration times and the peak resolutions for all analytes. In order to achieve the best separation of the investigated compounds and meet the requirement of accurate quantification in real samples, some important parameters that can influence the MEKC performance, including the effects of SDS, organic solvents, [bmim]BF_4_, β-CD, and buffer pH, were further optimized in the present study.

#### 2.2.1. Effect of SDS

**Figure 2:**
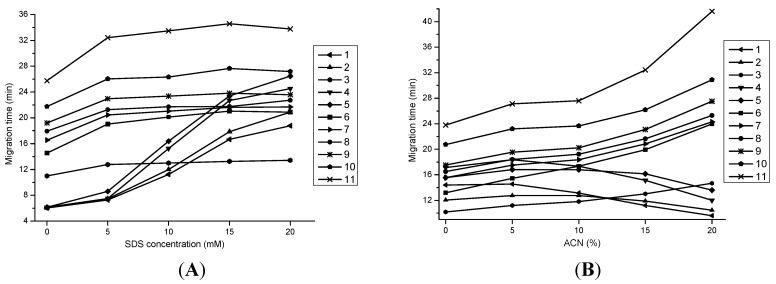
Effects of SDS and ACN concentration on migration behavior of the investigated analytes. Conditions: (**A**) 15 mM sodium tetraborate–5 mM β-CD–10 mM [bmim]BF_4_ containing 15% ACN (*v/v*) at pH 9.8 with different concentrations of SDS; (**B**) 15 mM sodium tetraborate–10 mM SDS–5 mM β-CD–10 mM [bmim]BF_4_ at pH 9.8 with different ratios of ACN.

In MEKC, SDS micelles act as a pseudo-stationary phase, which plays the key role in the separation of investigated analytes, especially lipophilic analytes. Different concentrations of SDS (0, 5, 10, 15, and 20 mM) were tested to separate a standard mixture, and the results are shown in Figure 2A. It was found that the addition of SDS had little effect on the separation of the seven hydrophilic analytes. In the range of 0–10 mM SDS, they were well separated from each other. For the four lipophilic compounds, they could not be separated from each other without the addition of SDS. When SDS was added to the buffer in the range of 10–15 mM, a remarkable separation was observed, suggesting that adequate SDS micelles had formed and interacted with the target components. When the SDS concentration was increased to 20 mM, the peaks of cryptotanshinone and salvianolic acid C partially overlapped. On the other hand, the migration times of the four lipophilic analytes became longer with increasing SDS concentrations. This may be because the hydrophobicity of the inner core of the SDS micelles was getting stronger under a higher concentration, leading to increased partitioning of the lipophilic analytes into the micellar phase. Finally, 10 mM was selected as the optimal concentration of SDS for subsequent studies since it could obtain the shortest migration time with a baseline separation of all 11 analytes.

#### 2.2.2. Effect of Organic Solvents

The previous work found that two pairs of peaks, namely tanshinone IIA and rosmarinic acid, and tanshinone I and danshensu, could not be separated from each other, even with the addition of [bmim]BF_4_ and β-CD. Therefore, different types of organic solvents were tested as additives in order to improve the separation, including MeOH, EtOH, ACN, and isopropanol. With the addition of ACN, the shortest analysis time and baseline separation of the targeted analytes could be obtained. Different ratios of ACN (0%, 5%, 10%, 15%, and 20%) were further investigated and the results are shown in Figure 2B. It can be observed that the migration times of the seven hydrophilic compounds were increasing with increasing ratios of ACN in the range of 0%–20%. Meanwhile, the migration times of the four lipophilic analytes showed the same increasing trend in 0%–5% ACN. However, when the ratios of ACN increased from 5% to 20%, a decreasing trend of migration times was noticed for the four lipophilic compounds. More importantly, only when using 15% ACN, a good baseline separation for all 11 compounds could be achieved. Thus, 15% ACN was adopted as the optimal ratio.

#### 2.2.3. Effect of [bmim]BF_4_

**Figure 3:**
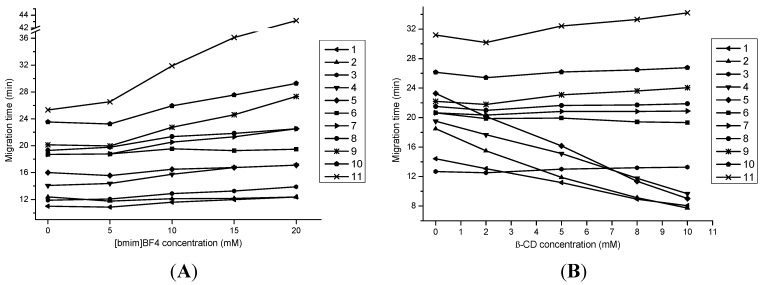
Effects of [bmim]BF_4_ and β-CD concentration on migration behavior of the investigated analytes. Conditions: (**A**) 15 mM sodium tetraborate–10 mM SDS–5 mM β-CD containing 15% ACN (*v*/*v*) at pH 9.8 with different concentrations of [bmim]BF_4_; (**B**) 15 mM sodium tetraborate–10 mM SDS–10 mM [bmim]BF_4_ containing 15% ACN (*v*/*v*) at pH 9.8 with different concentrations of β-CD.

In order to further enhance the selectivity for the investigated analytes, especially for lipophilic compounds, different concentrations of [bmim]BF_4_ (0, 5, 10, 15, 20 mM) were tested. As shown in Figure 3A, without the addition of [bmim]BF_4_, the peaks of cryptotanshinone and protocatechuic aldehyde were partially overlapped, as well as salvianolic acid C and rosmarinic acid, which showed only one single peak. When the concentration of [bmim]BF_4_ increased to 5 mM, the peak resolution of cryptotanshinone and protocatechuic aldehyde got more worse than that of no [bmim]BF_4_ addition. Meanwhile, the resolution of 9″-Methyl lithospermate B and danshensu decreased. Fortunately, once the concentration of [bmim]BF_4_ was up to 10 mM, all 11 analytes could be well separated with high resolution. However, when the concentration of [bmim]BF_4_ increased from 10 to 20 mM, more overlapping peaks appeared, accompanied with the deterioration of some peak shapes. Therefore, 10 mM [bmim]BF_4_ was accepted as the best ionic liquid additive.

#### 2.2.4. Effect of β-CD

Different concentrations of β-CD (2, 5, 8, and 10 mM) were added to the buffer solution to improve the MEKC separation. As indicated in Figure 3B, salvianolic acid C and rosmarinic acid could not be separated in the absence of β-CD. When 2 mM β-CD was added, the peaks of salvianolic acid C, rosmarinic acid and tanshinone IIA overlapped with low resolution. Fortunately, with the addition of 5 mM β-CD, all 11 components could achieve baseline separation and obtain high resolutions. Further, when the concentration of β-CD increased higher than 5 mM, the resolutions of the four lipophilic components showed a downward trend, whereas the separation of the seven hydrophilic analytes was not affected. Therefore, 5 mM β-CD offered the best separation efficiency for all the 11 analytes and therefore used in this study.

In addition, as shown in Figure 3B, the migration times of the four lipophilic components decreased with the increase of β-CD concentrations. A possible explanation is that, without the addition of β-CD, the separations of the four lipophilic compounds were mainly based on the partitioning between the SDS micellar phase and the aqueous phase. After the addition of β-CD, the four lipophilic compounds were partitioned into three phases, the SDS micellar phase, the aqueous phase and the β-CD phase, because β-CD has a lipophilic cavity that could dissolve lipophilic compounds. Since the surface of SDS micelles was negatively charged when the buffer pH was adjusted to 9.8, their electrophoresis migration was towards the opposite direction of EOF. On the contrary, the uncharged β-CD migrated with the EOF in the run buffer. Therefore, with the increasing concentration of β-CD, the partitioning of the four lipophilic compounds into the β-CD phase was getting stronger compared to SDS micellar phase, resulting in their decreased migration times.

#### 2.2.5. Effect of Buffer pH

Since the pH value of run buffer is a key parameter for analyte separation in MEKC mode, the effect of buffer pH was investigated by varying it from 8.5 to 10.3 to separate a mixed standard. The result showed that, at pH 8.5–9.5, the investigated analytes produced, not only a few overlapping peaks, but unacceptable poor peak shapes. When pH increased from 9.8 to 10.3, all 11 compounds could be separated, however, the peak resolution of salvianolic acid C and rosmarinic acid were getting worse with longer analysis times. Considering the highest selectivity and the shortest analysis time, the run buffer was adjusted to pH 9.8 in this study.

Considering the above-mentioned procedure, the optimum MEKC conditions were as follows: BGE, 15 mM sodium tetraborate with 10 mM SDS, 5 mM β-CD, 10 mM [bmim]BF_4_ and 15% ACN (*v*/*v*) as additives; buffer pH, 9.8; voltage, 20 kV; temperature, 25 °C; detection wavelength, 254 nm. Under the optimized conditions, the 11 investigated analytes could achieve baseline separation with 34 min.

### 2.3. Method Validation

The proposed MKEC method was validated by studying the linearity, limit of detection (LOD), limit of quantification (LOQ), precision and accuracy.

#### 2.3.1. Linearity, LOD and LOQ

The calibration curves of the 11 compounds were prepared by diluting appropriate volumes of a mixed standard solution with 70% MeOH into seven different concentrations as working solutions. All the measurements were determined in triplicate, depending on the electrophoretic procedure described in Section 2.3. The calibration curves were constructed by plotting the integrated peak areas of the analytes (*y*) *vs.* the corresponding concentrations (*x*). The standard curves, linear ranges, and correlation coefficients (R^2^) are calculated and listed in Table 1. 

**Table 1:** Calibration curves, limits of detection (LODs) and limits of quantification (LOQs) of the investigated compounds.

Peak No.	Analyte	Standard Curve ^a^	R^2^	Linear Range (μg·mL^−1^)	LOD (μg·mL^−1^)	LOQ (μg·mL^−1^)
1	Dihydrotanshinone I	*y* = 64*x* + 680	0.9967	1.53–61.25	0.27	0.90
2	Cryptotanshinone	*y* = 52*x* + 425	0.9968	2.50–100.00	0.42	1.42
3	Protocatechuic aldehyde	*y* = 36*x* + 745	0.9979	2.13–85.00	0.40	1.34
4	Tanshinone I	*y* = 37*x* + 603	0.9994	2.00–80.00	0.41	1.36
5	Tanshinone IIA	*y* = 35*x* + 1214	0.9988	4.95–198.00	0.67	2.24
6	Salvianolic acid C	*y* = 39*x* + 222	0.9981	1.51–121.00	1.39	4.63
7	Rosmarinic acid	*y* = 41*x* + 785	0.9965	4.31–345.00	1.30	4.34
8	9″-Methyl lithospermate B	*y* = 28*x* + 238	0.9973	2.50–100.00	1.17	3.91
9	Danshensu	*y* = 11*x* + 61	0.9993	2.95–118.00	1.31	4.33
10	Salvianolic acid B	*y* = 50*x* + 3351	0.9969	12.50–1000.00	1.10	3.67
11	Protocatechuic acid	*y* = 45*x* + 356	0.9987	2.75–110.00	0.93	3.09

^a^ *y* is the peak area and *x* is the corresponding injection concentration, respectively.

The values of R^2^ were higher than 0.9965 for all the compounds, indicating good linearity. Under optimal conditions, the LODs and LOQs were determined by measuring a series of decreasing standard solutions, based on a signal to noise (S/N) ratio of 3:1 for LOD and 10:1 for LOQ, respectively. The detailed data were shown in Table 1.

#### 2.3.2. Precision

The RSDs of migration time and peak area were taken as a measure of precision for the proposed MEKC method. For intra-day precision, the mixed standard solution was determined on five replicates within one day, while the inter-day precision was performed on five consecutive days.

The results (as indicated in Table 2) showed that the RSD values of migration time and peak area of the 11 analytes were all lower than 1.79% and 4.09% for the intra-day precision, 2.60% and 4.74% for the inter-day precision, respectively, revealing the good reproducibility of the MEKC method.

**Table 2:** Precision test of the optimized MEKC method (*n* = 5).

Analyte	Concentration (μg·mL^−1^)	Intra-Day RSD (%)		Inter-Day RSD (%)	
		Migration Times	Peak Areas	Migration Times	Peak Areas
Dihydrotanshinone I	61.25	0.98	2.99	0.66	3.44
Cryptotanshinone	100.00	0.94	2.14	0.53	3.15
Protocatechuic aldehyde	85.00	0.75	3.33	0.94	3.64
Tanshinone I	80.00	0.89	4.09	0.85	4.74
Tanshinone IIA	198.00	0.81	3.86	0.81	3.88
Salvianolic acid C	121.00	1.01	2.35	1.64	2.78
Rosmarinic acid	345.00	0.99	3.54	1.47	1.79
9″-Methyl lithospermate B	100.00	1.32	2.41	1.84	2.77
Danshensu	118.00	1.22	3.93	1.72	2.88
Salvianolic acid B	1000.00	1.79	1.03	2.18	3.72
Protocatechuic acid	110.00	1.62	3.08	2.60	3.83

#### 2.3.3. Accuracy

In order to evaluate the accuracy of the established MEKC method, the recovery study was carried out by spiking accurate amounts of the 11 standard solutions to a real sample (sample 3, *S. miltiorrhiza*, 1.00 g) with known contents of the target components in triplicate at three different levels (low, medium, high). The spiked samples were pretreated according to the method described in Section 3.4 and then measured by the procedure mentioned in Section 3.3. The recovery values (%) were calculated by the following equation:

(1) Recovery (%) = 100 × (founded amount − original amount)/added amount

As a result, the average recoveries for all 11 compounds were in the range of 94.26%–105.17% with RSDs less than 4.96%.

### 2.4. Application to Real Samples

**Figure 4:**
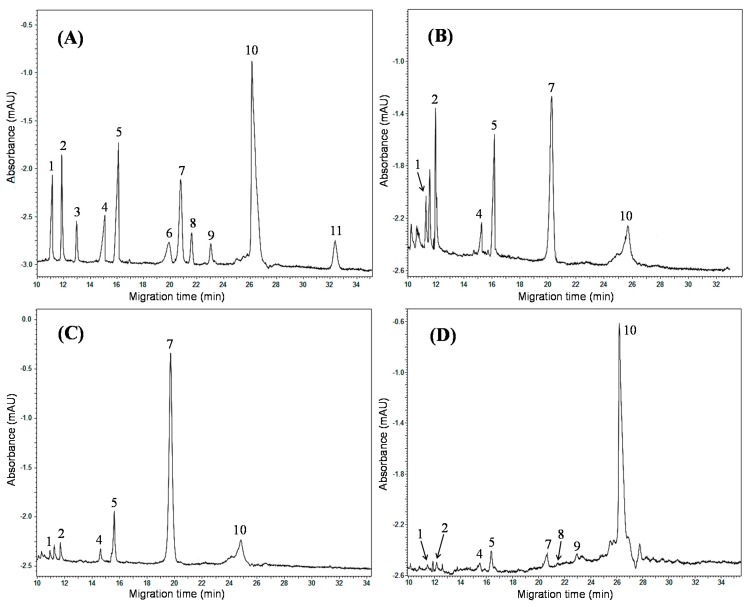
Typical electropherograms of the mixed standard solution and real samples under the optimal separation conditions. (**A**) Standard mixture (the concentrations are listed in Table 3); (**B**) No. 1, *S. przewalskii*; (**C**) No. 2, *S. castanea*; (**D**) No. 3, *S. miltiorrhiza.*

As the MEKC method has been demonstrated to be reliable and accurate, it was applied to quantitative analysis of the 11 investigated components in the roots of three *Salvia* plants of different origins. Typical electropherograms of the mixed standard and samples are shown in Figure 4. The identification of the target analytes was carried out by comparing their migration times with those of corresponding standards and also by spiking individual standards into the sample solutions. As shown in Figure 4, the mixed standard solution and the sample solutions of three different *Salvia* plants can be well resolved with high resolutions. The contents of the investigated compounds were calculated and presented in Table 3. 

For the seven hydrophilic phenolic acids, it can be seen that salvianolic acid B was the most dominant component in all the *S. miltiorrhiza* samples, which was used as one of the markers for the quality control of danshen in Chinese Pharmacopoeia. Additionally, the content of rosmarinic acid could also be detected in all the samples, in the range of 0.47 to 19.57 mg·g^−1^. Moreover, it can be found that, among the different *Salvia* species, the contents of salvianolic acid B and rosmarinic acid varied dramatically. The contents of rosmarinic acid in *S. przewalskii* and *S. castanea* samples were much higher than in *S. miltiorrhiza* samples, whereas salvianolic acid B mainly existed in *S. miltiorrhiza* samples, which could be attributed to the species differences in biology and the climate variation of their growth origins. Furthermore, it should be noted that the other five phenolic acids were not detected in *S. przewalskii* and *S. castanea* samples, which reveals that it is reasonable to select *S. miltiorrhiza* as unique and the official origin of danshen in the Chinese Pharmacopoeia (edtion 2010) [16]. For the four lipophilic tanshinones, they could be quantitatively detected in all the samples except one *S. miltiorrhiza* sample from Anhui. Among the four tanshinones, tanshinone IIA was the most abundant in all samples, which was chosen as another indicator for the quality control of danshen in the Chinese Pharmacopoeia.

In addition, a comparison of the current work with some other methods for the analysis of *Salvia* species is presented in Table 4. Compared with the HPLC methods [15,32], the proposed MEKC method and MEEKC approach [30] possessed a shorter analysis time, indicating their high separation efficiency. Although the proposed method is not as sensitive as these LC-based methods, it showed comparable precision and accuracy. Meanwhile, the HPLC methods consumed large amounts of organic solvents (e.g., acetonitrile and methanol), while only a few volumes of run buffer with a low ratio of acetonitrile and/or 1-butanol were used to achieve the effective separation in the current work or in the MEEKC-based study. Moreover, compared with the MEEKC technique, the separation parameters of the proposed MEKC method were more easily optimized.

## 3. Experimental Section

### 3.1. Chemicals

Protocatechuic aldehyde (HPLC >99% pure), protocatechuic acid (HPLC >99% pure), danshensu (HPLC >98% pure), rosmarinic acid (HPLC >98% pure), cryptotanshinone (HPLC >98% pure) and tanshinone IIA (HPLC >98% pure) were obtained from Shanghai Winherb Medical Technology Co., Ltd. (Shanghai, China). Salvianolic acid B (HPLC ≥98% pure), salvianolic acid C (HPLC ≥98% pure), 9″-Methyl lithospermate B (HPLC ≥96% pure), tanshinone I (HPLC ≥99% pure) and dihydrotanshinone I (HPLC ≥98% pure) were purchased from Shanghai Tauto Biotech Co., Ltd. (Shanghai, China). 

Sodium tetraborate was supplied by Tianjin Damao Chemical Reagent Factory (Tianjin, China) and SDS was acquired from Beyotime Institute of Biotechnology (Haimen, China). The ionic liquid [bmim]BF_4_ and β-CD were purchased from Aladdin Reagent Co., Ltd. (Shanghai, China).

In this experiment, deionized water (18.2 MOhm-cm) was used and prepared by a Milli-Q water system (Millipore, Billerica, MA, USA). HPLC-grade ACN was bought from Merck Co. (Darmstadt, Germany). All other reagents and chemicals were of analytical grade.

The *Salvia* samples, *S. miltiorrhiza*, *S. przewalskii* and *S. castanea*, were collected from different provinces in China. All the samples were authenticated by Shimin Guo at the Yunnan Institute of Traditional Chinese Medicine and Medical Materials, Kunming, China.

**Table 3:** Contents (mg·g^−1^) of 11 compounds in samples from different origins ^a^.

No.	Samples	Origin	Dihydrotanshinone I	Cryptotanshinone	Protocatechuic Aldehyde	Tanshinone I	Tanshinone IIA	Salvianolic Acid C	Rosmarinic Acid	9″-Methyl lithospermate B	Danshensu	Salvianolic Acid B	Protocatechuic Acid
1	*S. przewalskii*	Yunnan	0.59 ± 0.07 ^b^	2.49 ± 0.18	-	0.87 ± 0.10	3.88 ± 0.12	-	12.51 ± 1.27	-	-	4.97 ± 0.17	-
2	*S. castanea*	Yunnan	0.14 ± 0.01	0.50 ± 0.06	-	0.47 ± 0.04	2.84 ± 0.12	-	19.57 ± 1.03	-	-	2.43 ± 0.09	-
3	*S. miltiorrhiza*	Shandong	0.09 ± 0.01	0.19 ± 0.03	-	0.18 ± 0.13	0.60 ± 0.19	-	0.47 ± 0.16	+	1.97 ± 0.65	16.47 ± 1.57	-
4	*S. miltiorrhiza*	Sichuan	0.07 ± 0.02	0.32 ± 0.11	0.55 ± 0.06	0.16 ± 0.01	1.84 ± 0.20	0.13 ± 0.02	1.86 ± 0.36	0.26 ± 0.04	0.52 ± 0.11	44.75 ± 1.29	-
5	*S. miltiorrhiza*	Anhui	-	-	-	-	+	-	1.48 ± 0.37	-	0.66 ± 0.07	25.95 ± 1.21	-
6	*S. miltiorrhiza*	Hebei	0.25 ± 0.05	0.46 ± 0.05	0.10 ± 0.01	0.52 ± 0.11	1.68 ± 0.14	-	0.97 ± 0.20	0.21 ± 0.03	0.40 ± 0.05	17.77 ± 0.79	-
7	*S. miltiorrhiza*	Hebei	0.14 ± 0.04	0.34 ± 0.05	-	0.23 ± 0.03	0.57 ± 0.08	-	1.40 ± 0.06	0.28 ± 0.05	0.50 ± 0.09	24.91 ± 2.15	-

^a^ Data are presented as the average of triplicate. ^b^ Mean ± S.D.; “-” not detected; “+” under the limits of quantification.

**Table 4:** Comparison of current work and some other reports.

Matrix	Analytes	Detection Method	Analysis Time (min)	Solvent Consumption	LOD (μg·mL^−1^) ^a^	Inter-Day RSD (%)	Recovery	Ref.
*S. miltiorrhiza*, *S. przewalskii*, *S. castanea*	1–11	MEKC	34	<1 mL	1.10	≤4.74	94.3%–105.2%	Current work
*S. miltiorrhiza*	1–3, 5–9, 11, miltirone, methylene tanshiqunone, caffeic acid, lithospermic acid	MEEKC	30	<1 mL	0.44	N/A	N/A	[30]
*S. miltiorrhiza*	1–7, 9–11, miltirone, methylene tanshiqunone, caffeic acid, lithospermic acid, salvianolic acid A	HPLC-UV	70	~70 mL	0.09	≤2.00	95.0%–103.7%	[15]
*S. miltiorrhiza* and its preparation	1, 2, 4, 5, 10	HPLC-DAD	45	~45 mL	0.14	≤3.94	97.9%–105.3%	[32]
*S. miltiorrhiza*	1–5, 7, 10, 11, caffeic acid, ferulic acid, isoferulic acid, salvianolic acid A, przewalskin	UPLC-DAD	16	~8 mL	0.35	≤4.85	91.1%–104.2%	[20]

^a^ Data are presented as the LOD value of salvianolic acid B.

### 3.2. Apparatus

All MEKC experiments were performed on a Beckman P/ACE MDQ capillary electrophoresis system equipped with an auto sampler and a photo diode array (PDA) detector (Beckman coulter, Fullerton, CA, USA). 32 Karat system version 8.0 (Beckman coulter, Fullerton, CA, USA) was used for data acquisition and processing. The separations were carried on uncoated fused-silica capillaries with 50-cm effective length, 75 μm I.D., and 365 μm O.D. (Ruifeng, Handan, China). The pH of BGE was adjusted by a SevenEasy pH-meter (Mettler Toledo, Greifensee, Switzerland) with 1.0 M NaOH or 1.0 M HCl. Both sample extraction and solution degassing were performed on an ultrasonic cleaner (Branson Ultrasonic Corp., Danbury, CT, USA).

### 3.3. Electrophoretic Procedure

The run buffer of this MEKC experiment was 15 mM sodium tetraborate–10 mM SDS–5 mM β-CD–10 mM [bmim]BF_4_ containing 15% ACN (*v*/*v*) (pH 9.8). The separation voltage and capillary temperature were set at 20 kV and 25 °C, respectively. Samples and standard solutions were injected into the capillary column under a pressure of 0.3 psi for 2 s. The detection wavelength was 254 nm.

Prior to its daily use, the capillary was rinsed with 0.1 M NaOH for 10 min, followed by purified water for 5 min, and finally conditioned with run buffer for 5 min. Before each run, the capillary was sequentially rinsed with 0.1 M NaOH for 2 min, purified water for 2 min, and finally run buffer for 4 min. Vials with run buffer (inlet and outlet) were replaced every two injections to avoid the changes of EOF due to the electrolysis of buffer solutions under a high voltage. All the solutions were filtered through a 0.22 μm membrane and degassed by sonication before use.

### 3.4. Sample Preparation and Standard Solutions

An aliquot of 2.00 g sample powder (through 50 mesh screen) was accurately weighed and extracted by sonication for 30 min after addition of 50 mL methanol/water (70/30, *v*/*v*). The solution was then adjusted to its original weight with extraction solvents. The extract was centrifuged at 4700× *g* for 15 min and the supernatant was filtered through a 0.22 μm membrane (Millex-GV, 13 mm, Millipore) for MEKC analysis.

Individual stock solutions of the 11 reference standards were prepared in methanol at the concentration ranging from 1.25 to 10.00 mg·mL^−1^. The stock standard mixture was obtained by dissolving appropriate amounts of the individual stock solutions with methanol/water (70/30, *v*/*v*) to a desired concentration. All the solutions were stored at 4 °C prior to analysis.

## 4. Conclusions

In the current work, a novel MEKC method has been developed to simultaneously separate seven hydrophilic and four lipophilic bioactive compounds in the roots of *Salvia* plants. In order to achieve the best separation of the investigated compounds, the effects of SDS, additives (including organic solvents, [bmim]BF_4_, β-CD), and buffer pH on the MEKC performance have been explored. Further validation study demonstrated that the newly proposed MEKC method was reproducible, accurate, and reliable. Under optimized conditions, the method was successfully applied for the analysis of 11 analytes in three *Salvia* species, which exhibited strong separation power and good applicability for these real samples. In short, the established MEKC method was simple and practical for the simultaneous determination of the hydrophilic and lipophilic bioactive components, and could be used to effectively evaluate the quality of *Salvia* plants.
